# The Influence of E1A C-Terminus on Adenovirus Replicative Cycle

**DOI:** 10.3390/v9120387

**Published:** 2017-12-19

**Authors:** Leandro Crisostomo, Andrea Michelle Soriano, Jasmine Rae Frost, Oladunni Olanubi, Megan Mendez, Peter Pelka

**Affiliations:** 1 Department of Microbiology, University of Manitoba, 45 Chancellor’s Circle, Buller Building Room 427, Winnipeg MB R3T 2N2, Canada; umcrisol@myumanitoba.ca (L.C.); sorianoa@myumanitoba.ca (A.M.S.); umfrost3@myumanitoba.ca (J.R.F.); oladunniolanubi@live.com (O.O.); megan.mendez@umanitoba.ca (M.M.); 2 Department of Medical Microbiology, University of Manitoba, Winnipeg MB R3E 0J9, Canada

**Keywords:** E1A, C-terminus, adenovirus

## Abstract

Adenovirus Early 1A proteins (E1A) are crucial for initiation of the viral life cycle after infection. The *E1A* gene is encoded at the left end of the viral genome and consists of two exons, the first encoding 185 amino acids in the 289 residues adenovirus 5 E1A, while the second exon encodes 104 residues. The second exon-encoded region of E1A is conserved across all E1A isoforms except for the 55 residues protein, which has a unique C-terminus due to a frame shift following splicing into the second exon. This region of E1A contributes to a variety of processes including the regulation of viral and cellular gene expression, immortalization and transformation. Here we evaluated the contributions that different regions of the second exon of E1A make to the viral life cycle using deletion mutants. The region of E1A encoded by the second exon was found to be important for overall virus growth, induction of viral and cellular gene expression, viral genome replication and deregulation of the cell cycle. Efficient viral replication was found to require exon 2 and the nuclear localization signal, as loss of either resulted in severe growth deficiency. Induction of cellular DNA synthesis was also deficient with any deletion of E1A within the C-terminus even if these deletions were outside of conserved region 4. Overall, our study provides the first comprehensive insight into the contributions of the C-terminus of E1A to the replicative fitness of human adenovirus 5 in arrested lung fibroblasts.

## 1. Introduction

Human adenovirus (HAdV) usually infects terminally differentiated epithelial cells [1]. Since this environment is unsuitable for replication, the immediate early E1A proteins reprogram the infected cell to facilitate viral replication, which involves induction of the cell cycle and entry of the infected cells into the S-phase to allow viral DNA to be copied [1]. E1A is a small protein of 289 residues (R) in the largest isoform of HAdV5. E1A is encoded by the *E1A* gene; the pre-mRNA is spliced into five different splice variants that are expressed differentially during the course of viral infection [2]. The largest isoforms of E1A, derived from the 13S and 12S mRNAs, are most abundant early in infection, while the smaller isoforms become more abundant once viral genomes begin replicating with the 10S mRNA and the derived 171R protein being the most abundant E1A during late infection [2].

The *E1A* gene is composed of two exons that splice alternatively to give the five different protein isoforms. The first exon undergoes additional alternative splicing, removing much of conserved region (CR) 1, to generate the later E1A mRNAs found in infection [2]. The functions that E1A performs are largely executed via a large variety of protein-protein interactions between E1A and cellular factors [3]. Some of these disrupt interactions between cellular proteins while others, likely form de novo interactions that alter protein function either directly or by inducing novel post-translational modifications via bridging enzymes with novel targets. Most of the interactions between E1A and cellular proteins have been described for the region of E1A encoded by exon 1, while exon 2 encoded region (henceforth referred to as the C-terminus and consisting of amino acids 186–289 in HAdV5 E1A289R), despite contributing a substantial number of amino acids to E1A, has been poorly studied [3]. Until recently, only a handful of C-terminus binding partners have been described [4], including the C-terminus Binding Protein (CtBP) [5], FOXK1 [6], importin α3 [7] and DYRK1A [8]. Recently, our group has identified three new E1A C-terminus binding partners; DREF that functions in innate immunity and whose SUMOylation is altered by E1A [9], Ku70 that appears to be important for the inhibition of the DNA damage response pathway during infection [10] and RuvBL1 that plays an important role in E1A-mediated suppression of type I interferon pathway [11]. Despite these recent advances in our understanding of the contribution of the region of E1A encoded by exon 2, we still lack a complete picture of how this region contributes to the viral life cycle in normal human cells.

In the present study, we undertook the examination of how small deletions within the exon 2 of E1A affect viral fitness during infection of normal lung WI-38 fibroblasts that have been arrested by contact inhibition. Deletions of small regions of exon 2 varied greatly in the effect they had on virus growth, viral gene and protein expression, viral genome replication and modulation of the cell cycle. All mutant viruses were deficient in growth as compared to HAdV5 expressing wild-type (wt) E1A (*dl*309). Interestingly, deletion of the extreme C-terminus including a portion of the bipartite nuclear localization signal (NLS) [12] had the most profound effect on virus growth, second only to the loss of entire exon 2. Our study provides the first comprehensive analysis of how the different regions of E1A C-terminus contribute to the viral replicative cycle in arrested human cells, the natural target of the virus.

## 2. Materials and Methods

### 2.1. Antibodies

As performed previously [2], mouse monoclonal anti-E1A M58 antibody was previously described [13] and was grown in-house and used as the supernatant of M58 hybridoma cells. The M58 antibody binds within the region encoded by the first exon of E1A (between residues 63 and 99 of E1A) and recognizes all mutant E1A proteins used in this study [14]. Mouse monoclonal anti-72k DNA binding protein (DBP) antibody was previously described [15] and was used at a dilution of 1:400 for western blot. Anti-adenovirus type 5 antibody was purchased from Abcam (catalog number ab6982, Cambridge, MA, USA). Actin antibody was purchased from Abcam (catalog number ab3280). Secondary antibodies were acquired from Jackson ImmunoResearch (West Grove, PA, USA) and were used at a dilution of 1:200,000.

### 2.2. Cell and Virus Culture

As carried out previously [2], 293 (ATCC CRL-1573) and WI-38 (ATCC CCL-7) cells were grown in Dulbecco’s modified Eagle’s medium (HyClone, Logan, UT, USA) and supplemented with 10% fetal bovine serum (VWR Seradigm, Mississauga, ON, Canada) and streptomycin and penicillin (HyClone). Cells were incubated at 37 °C with 5% CO_2_. All virus infections were carried out in serum-free media for 1 h, after which saved complete media was added without removal of the infection media. All viruses used were of the same genetic background as *dl*309; i.e., all mutants and *dl*309 have the same deletion in the E3 region. For all infections, titered crude freeze-thaw lysates were used.

### 2.3. EdU Incorporation Assay

As described [2], WI-38 cells were grown until 100% confluent on LabTek II 4-chamber slides (Thermo-Fisher, Waltham, MA, USA). After becoming fully confluent, cells were incubated for a further 72 h to achieve growth arrest. Infections were carried out as described above with a multiplicity of infection (MOI) of 100 for *dl*309 [16], or *dl*311 [16,17], or *dl*1116 [18], or *dl*1132 [19], or *dl*1133 [5], *dl*1134 [5], or *dl*1135 [5], or *dl*1136 [5]. One hour prior to fixation, cells were pulsed with 5-ethynyl-2´-deoxyuridine (EdU) for 1 h as per manufacturer’s specifications using the Click-It EdU labeling kit for microscopy (Life Technologies, Carlsbad, CA, USA). After EdU labeling, cells were fixed in 3.7% formaldehyde, stained for EdU using the Click-It kit with AlexaFluor 488 and labelled for E1A using M58 monoclonal antibody and AlexaFluor 594 conjugated secondary anti-mouse antibody (Jackson ImmunoResearch). Cells were visualized using LSM700 laser confocal microscope (Carl Zeiss AG, Oberkochen, Germany) and ZEN software suite (Carl Zeiss AG, Oberkochen, Germany).

### 2.4. Immunofluorescence

As performed and described previously [10], WI-38 cells were plated at low density (40,000 cells per chamber) on chamber slides (Nalgene Nunc, Rochester, NY, USA) and subsequently infected as described above. Twenty-four hours after infection, cells were fixed in 4% formaldehyde, blocked in blocking buffer (1% normal goat serum, 1% bovine serum albumin, 0.2% Tween-20 in phosphate buffered saline) and stained with specific primary antibodies. M58 was used neat (hybridoma supernatant) and AlexaFluor 488 secondary antibody (Jackson ImmunoResearch) was used at a dilution of 1:600. After staining and extensive washing, slides were mounted using Prolong Gold with 4′,6-diamidino-2-phenylindole (DAPI) (Invitrogen, Carlsbad, CA, USA) and imaged using Zeiss LSM700 confocal laser scanning microscope. Images were analyzed using Zeiss ZEN software package.

### 2.5. PCR Primers

All primers used were previously described [2,10]. All primers were purchased from Integrated DNA Technologies (Coralville, IA, USA) and annealing temperature of 60 °C was used.

### 2.6. Real-Time Gene Expression Analysis

As performed previously [2], arrested WI-38 cells were infected with the different viruses at a MOI of 100 for 16, 24, 48 and 72 h. Total RNA was extracted using the TRIzol Reagent (Sigma, St. Louis, MO, USA) at the indicated time points according to manufacturer’s instructions. 1.25 μg of total RNA was used in reverse-transcriptase reaction using SuperScript VILO reverse transcriptase (Invitrogen) according to the manufacturer’s guidelines using random hexanucleotides for priming. The cDNA was subsequently used for real-time expression analysis using the BioRad CFX96 real-time thermocycler (BioRad, Hercules, CA, USA). Analysis of expression data was carried out using the Pfaffl method [20] and was normalized to glyceraldehyde 3-phosphate dehydrogenase (GAPDH) mRNA levels; these were compared to *dl*309 for viral gene analysis and to mock infected samples for cellular genes analysis.

### 2.7. Statistical Analysis

Statistics were performed as described by [21]. Briefly, statistical analysis was conducted using one-way analysis of variance (ANOVA) followed by post hoc comparison using Tukey test of cellular and viral genes from *dl*309 infection versus mutant virus infection. Viral growth assays and genome quantification assays were also subjected to ANOVA with post hoc comparison using Tukey test comparing mutants to *dl*309. *p*-Values were two-tailed and values of <0.05 were considered statistically significant in gene expression assays, viral growth assays and genome quantification assays. Student’s independent sample t-test was conducted on EdU incorporation assays. *p*-Values were one-tailed and values of <0.05 were considered statistically significant in the EdU incorporation assays.

### 2.8. Viral Genome Quantification

As we have done previously [2], arrested WI-38 cells were infected with the different viruses at a MOI of 100. The cells were infected for 24, 48 and 72 h and were lysed in lysis buffer (50 mM Tris pH 8.1, 10 mM ethylenediaminetetraacetic acid and 1% sodium dodecyl sulphate (SDS)) on ice for 10 min. Lysates were sonicated briefly in a Covaris M220 focused ultrasonicator to break-up cellular chromatin and subjected to digestion, using Proteinase K (NEB) according to manufacturer’s specifications. Following digestion viral DNA was purified using GeneJET PCR Purification Kit (Thermo-Fisher). PCR reactions were carried out using SYBR Select Master Mix for CFX (Applied Biosystems, Foster City, CA, USA) according to manufacturer’s directions using 2% of total purified DNA as template using a CFX96 Real Time PCR instrument (BioRad). Standard curve for absolute quantification was generated by serially diluting pXC1 plasmid containing the left end of HAdV5 genome starting with a concentration of 1.0 × 10^7^ copies per reaction down to 1.0 copy per reaction. The primers used were the same as those used for expression analysis of E1B region; the annealing temperature used was 60 °C; and 40 cycles were run.

### 2.9. Virus Growth Assay

As previously described [2], arrested WI-38 cells were infected with the different viruses at MOI of 100 in serum-free medium. HAdV5 mutant *dl*309 deletes portion of the E3 region that is not necessary for growth in cell culture and all of the mutants used were generated in *dl*309 background. Virus was adsorbed for 1 h at 37 °C under 5% CO_2_, after which cells were bathed in conditioned media and were re-incubated at 37 °C under 5% CO_2_. Virus titers were determined at 48, 72 and 96 h after infection by plaque assays performed on 293 cells by serial dilution.

## 3. Results

### 3.1. Growth of C-Terminus Deletion Mutants in Arrested Fibroblasts

**Figure 1:**
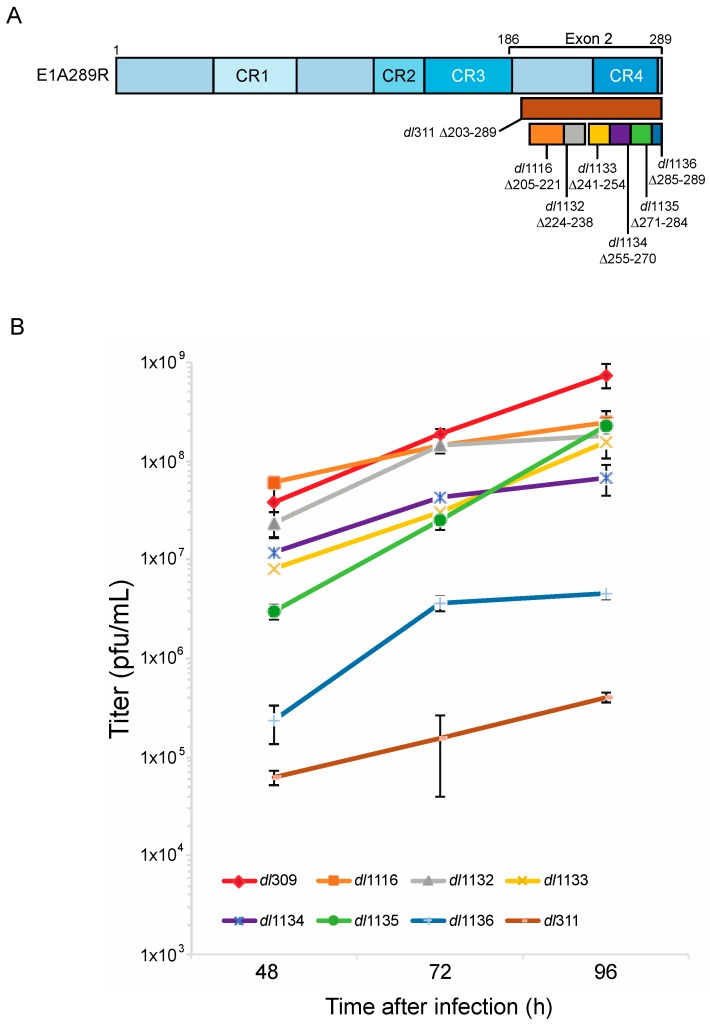
Deletions within the E1A C-terminus affect virus growth. (**A**) Schematic representation of HAdV5 E1A289R protein with locations of deletion mutants used in this study. Numbers indicate amino acids. (**B**) Contact inhibited WI-38 cells were infected with the indicated HAdV5 deletion mutants at a MOI of 100. Virus titers were determined at the indicated times points by performing plaque assays on 293 cells. At 48 h the differences in growth between *dl*309 and *dl*311, *dl*1135 and *dl*1136 were statistically significant with *p* < 0.0005, while others were not significant. At 72 h, the differences in growth between *dl*309 and *dl*1116 and *dl*1132 were not significant but were found to be statistically significant for all the other viruses with *p* < 0.0001. At 96 h, the differences in growth between *dl*309 and all the mutants tested were statistically significant with *p* < 0.0003. Error bars represent standard deviation of biological replicates, *n* = 3.

To test how deletions of E1A C-terminus affect virus growth in arrested WI-38 cells we used E1A mutants *dl*1116, *dl*1132, *dl*1133, *dl*1134, *dl*1135, *dl*1136 and *dl*311. Mutants *dl*1116 through *dl*1136 collectively delete subsets of residues 205 to 289 of E1A289R, while mutant *dl*311 deletes residues 203 to 289 (Figure 1). We initially determined the efficiency of infection of arrested WI-38 cells for each mutant at MOI of 10, 30 and 100 to determine the optimal MOI. Unexpectedly, we observed relatively low infectivity at MOI of 10 and 30 with approximately 30–50% of cells expressing E1A 24 h after infection at MOI of only 30 as judged by immunostaining. This was surprising as standard Poisson distribution would suggest nearly 100% efficiency of infection at MOI of 30 based on statistics alone. We therefore tested MOI of 100, which gave nearly 100% infection as judged by immunostaining for E1A at 24 h after infection. We chose this MOI for all subsequent experiments.

Virus growth was assayed at 48, 72 and 96 h after infection (Figure 1). No growth was observed at 24 h after infection, which was consistent with previous reports showing that in arrested cells virus does not begin to egress until after 24 h after initial infection [2,22]. *dl*311, which expresses E1A lacking most of the region encoded by exon 2, grew minimally throughout the assay duration. *dl*311 reached a titer of approximately 2E5 pfu/mL at 96 h, which is about 10,000× lower to the titer observed for *dl*309, expressing wt E1A and lower to what was previously observed for *dl*311 in transformed cells [16]. Greatest differences in virus growth were observed at 48 h after infection, with some viruses nearly reaching *dl*309 growth levels by 96 h or equalizing with other mutant viruses. Of all deletion mutants, *dl*1136 was the second worst replicating virus. It stalled at 72 h and never reached a titer of 1E7 pfu/mL, at least 10× lower than the next worst mutant but still much higher than *dl*311.

**Figure 2:**
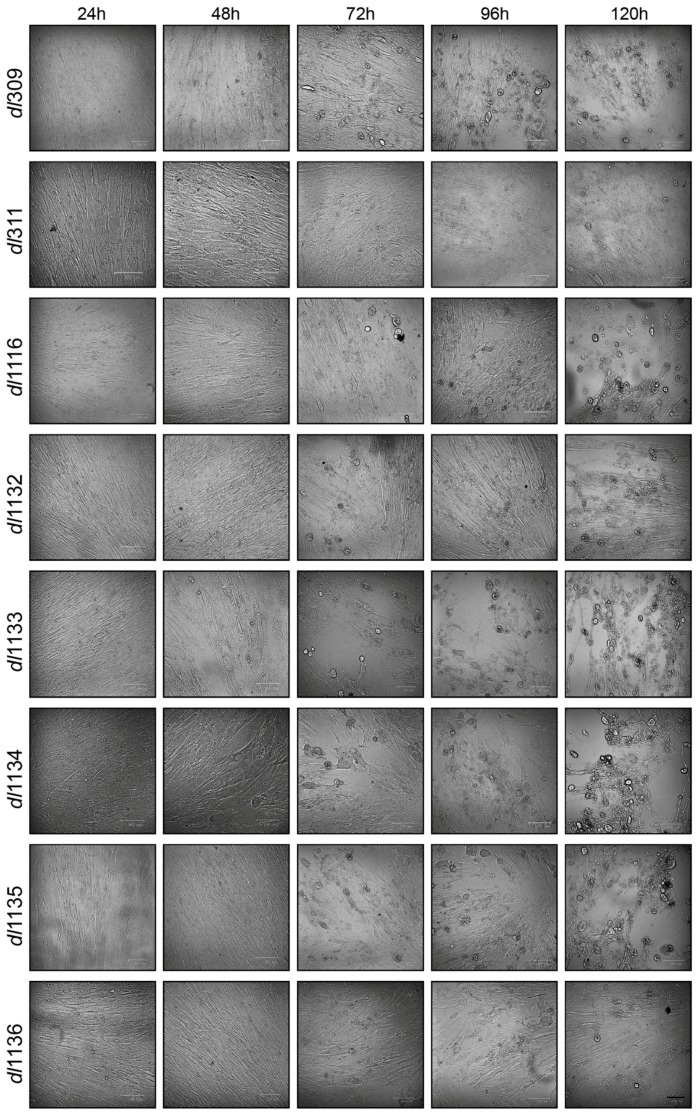
Deletions within the E1A C-terminus affect appearance of CPE. Arrested WI-38 cells were infected at a MOI of 100 and imaged at the indicated time points. Bar in lower right corner represents 100 µm, cells were all imaged using the same magnification (20× objective lens).

To assess how replication affected the morphology of the infected cells we examined cellular appearance by microscopy every 24 h after infection (Figure 2). Morphological changes associated with virus growth and replication were not observed for any virus at 24 after infection. Changes to cellular appearance and cytopathic effect (CPE) started to become apparent at 48 and 72 h after infection, respectively, for those cells infected with most viruses except for *dl*311 and *dl*1136, which looked normal with no cell enlargement or cellular detachment (Figure 2). CPE only became evident in *dl*1136 infected cells at 96 h after infection and only minor changes in cellular appearance were observed in cells infected with *dl*311 at 120 h after infection. Control uninfected cells were also monitored for any morphological changes over 120 h and showed no differences between the time of infection or 120 h later, consistent with the behaviour of arrested lung fibroblasts.

### 3.2. Viral Protein and Gene Expression

**Figure 3:**
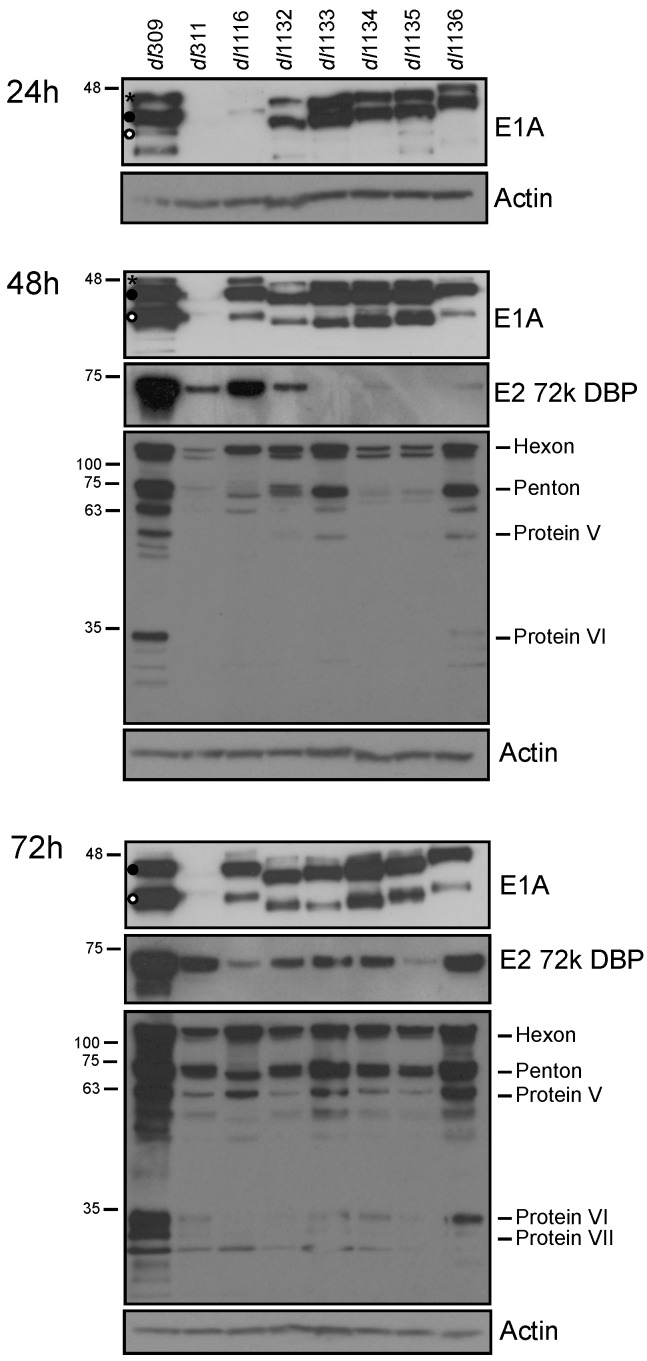
Viral protein levels in infected WI-38 cells. Arrested WI-38 cells were infected at a MOI of 100 and proteins were extracted at the indicated time points. Total cell lysate (20 µg) was resolved by SDS polyacrylamide gel electrophoresis, blotted for the indicated proteins and visualized using film. Actin was used as a loading control. * denotes E1A289R, • denotes E1A243R and ° denotes E1A171R.

**Figure 4:**
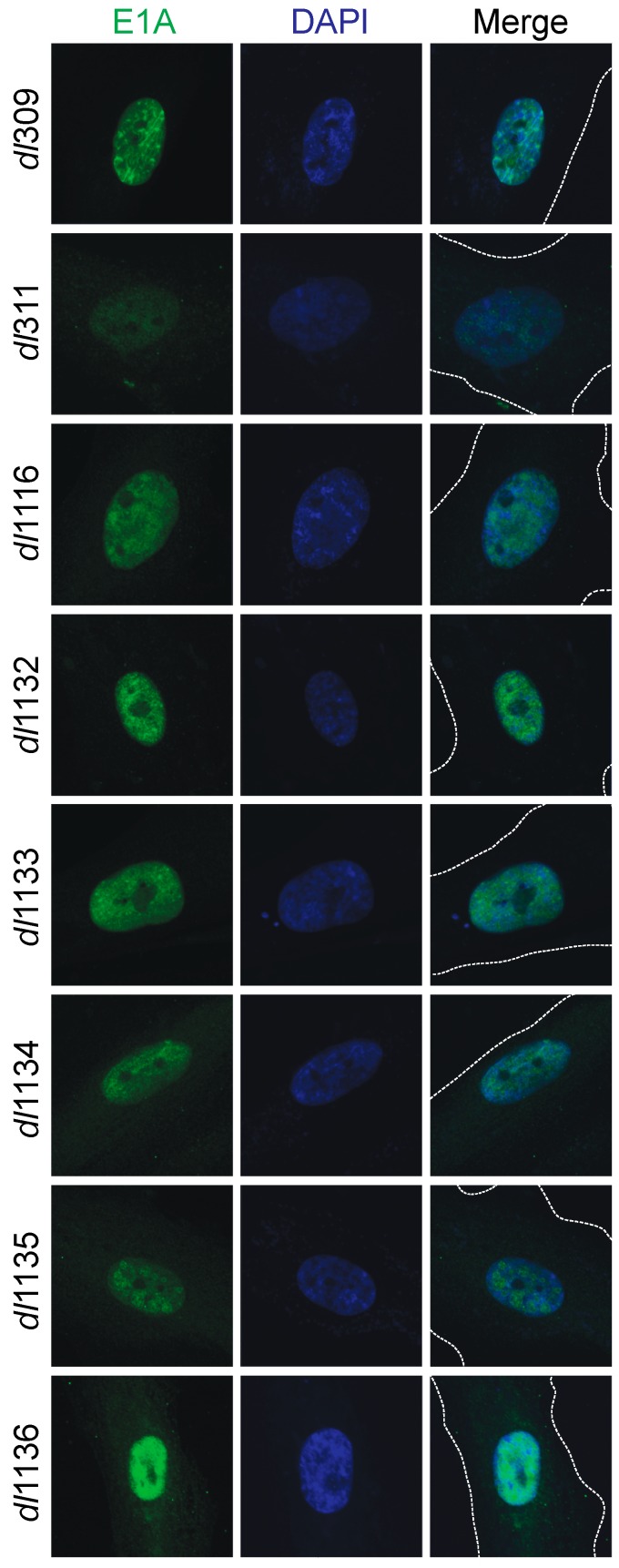
Sub-cellular localization of E1A and deletion mutants in infected cells. Arrested WI-38 cells were infected with the indicated mutants at a MOI of 100 for 24 h. Cells were then fixed, permeabilized and stained with anti-E1A antibody (M58) and a secondary anti-mouse AlexaFluor 488-conjugated antibody to visualize E1A. DAPI was used as a nuclear counterstain. The dashed lines in the merged image indicate cell boundaries. Images were acquired on a Zeiss LSM700 laser confocal microscope using the 63× objective lens.

To determine the effects of E1A deletions on viral protein expression, infected cells were lysed and western blot performed 24, 48 and 72 h after infection for E1A, E2 72k DBP and viral late proteins (Figure 3). E1A levels were readily detectable at 24 h after infection except for *dl*311 and *dl*1116. *dl*311 E1A was never observed by western blot, only by immunofluorescence (Figure 4). Levels of E1A continued to increase, with an observed shift in isoform abundance that we have previously observed [2]. Specifically, early in infection E1A243R was the most abundant isoform (denoted with • in Figure 3), E1A289R (denoted with * in Figure 3) overall was much less abundant with highest levels observed at 24 h after infection and nearly disappeared by 72 h (top-most band on E1A blots), whereas the 10S-derived E1A171R (denoted with ° in Figure 3) became more abundant later on, particularly in *dl*309-infected cells. DBP was only detectable at 24 h in *dl*309-infected cells and became more abundant at 48 h after infection for most of the mutants with the exception of *dl*1133 and *dl*1135. DBP protein levels increased during the course of the infection, with all infections showing readily detectable protein at the 72 h time point. Viral late proteins were not detected at 24 h after infection. Late proteins were readily detectable at 48 and 72 h after infection for all viruses except for *dl*311, which only showed these proteins weakly at this time point and readily at 72 h after infection. Interestingly, levels of late proteins did not correlate with virus growth, since *dl*311 grew very poorly yet had some late protein levels that were similar to other mutants at 72 h after infection, while *dl*1136 had relatively high late protein expression, second only to *dl*309, yet grew quite poorly when compared to other mutants (Figure 3).

**Figure 5:**
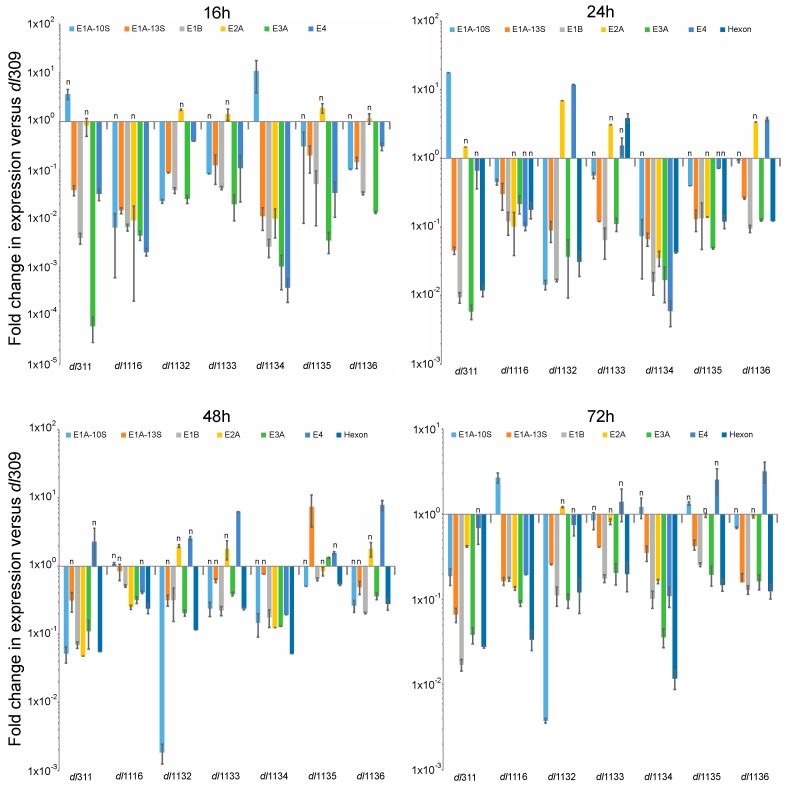
Relative viral gene expression profiles as compared to *dl*309. Arrested WI-38 cells were infected at a MOI of 100 and total RNA was extracted at the indicated time points using the TRIzol reagent and treated with DNase I. RNA was converted to cDNA using VILO Master Mix reverse transcriptase and relative levels of viral mRNAs were quantified by qPCR using BioRad CFX96 with Applied Biosystems SYBR Master Mix for CFX. Expression levels were compared to those obtained from *dl*309-infected cells using the Pfaffl method with GAPDH as a reference. Statistically insignificant differences are indicated with an *n*. The *p* values for all the genes at a time point that are significant are: 16 h—*p* < 0.023; 24 h—*p* < 0.0001; 48 h—*p* < 0.0063; 72 h—*p* < 0.0021. Error bars represent standard deviation of biological replicates, *n* = 3.

Expression of viral genes was determined at 16, 24, 48 and 72 h after infection by reverse-transcriptase quantitative real-time PCR (qPCR). Expression was directly compared to levels of transcripts present in *dl*309-infected cells at the same time point (Figure 5). We examined expression levels of the following mRNAs: E1A-10S, E1A-13S, E1B-55k, E2A, E3A, E4 orf6/7 and hexon. Most viral genes were expressed at much lower level in the mutants than in *dl*309 infected cells and this largely correlated with protein levels. For example, hexon protein was the highest in *dl*309, *dl*1133 and *dl*1136 and the hexon mRNA was also higher in the two mutants versus the remaining mutants that had lower hexon protein levels. Overall, the mutant viruses were approaching *dl*309 expression levels the longer the infection was allowed to proceed, since the differences contracted at 48 and 72 h as compared to 16 and 24 h for most viral genes.

### 3.3. Sub-Cellular Localization of Mutant E1A Proteins

We investigated the sub-cellular localization of the different mutant E1A proteins and how this compared to wt E1A (Figure 4). Arrested WI-38 cells were infected and stained for E1A 24 h later using the M58 antibody that would detect all mutants; DAPI was used as a nuclear counterstain. All E1A proteins showed nearly exclusive nuclear localization, with the exception of E1A *dl*311, E1A *dl*1134, E1A *dl*1135 and E1A *dl*1136, which showed more cytoplasmic E1A present but with the majority of the E1A protein still being localized to the nucleus. 

### 3.4. Viral Genome Replication

**Figure 6:**
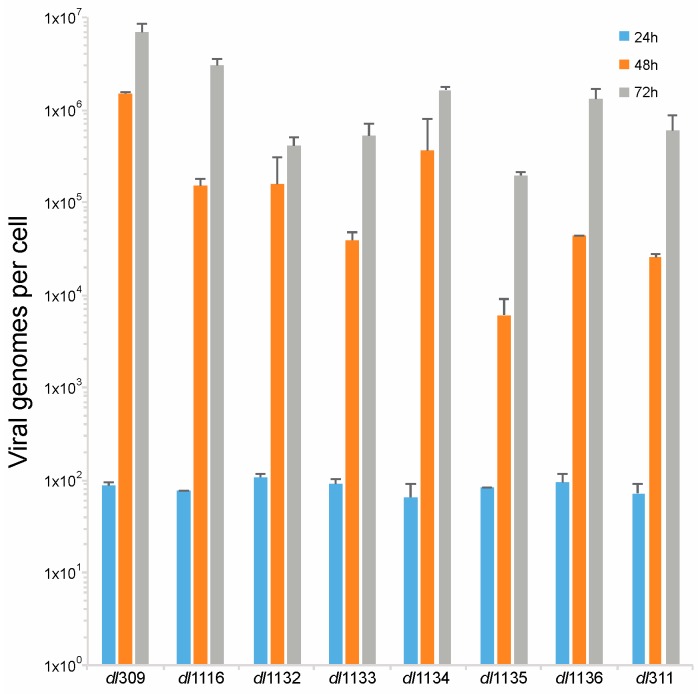
Deletions within the C-terminus of E1A affect viral genome replication. Arrested WI-38 cells were infected with the indicated mutant viruses at a MOI of 100. At the indicated time points, viral DNA was extracted and quantified by qPCR using the E1B primers and pXC1 plasmid to generate the standard curve. Viral genomes are plotted on per cell basis. All differences at 48 and 72 h were statistically significant versus *dl*309 with a *p* value ≤ 0.0002, except for the 24 h time point where there was no statistically significant difference between *dl*309 and the mutant viruses. Error bars represent standard deviation of biological replicates, *n* = 3.

E1A is crucial for induction of S-phase in arrested cells [23], which is essential for viral genome replication. Therefore, we investigated how deletions in the C-terminus of E1A affect viral genome replication. Arrested WI-38 cells were infected at a MOI of 100 and viral genomes were quantified at 24, 48 and 72 h after infection using qPCR. We did not observe any genome replication at 24 h, which is consistent with our prior observations that genome replication in arrested lung fibroblasts begins sometime between 24 and 48 h after infection (Figure 6) [2]. At 48 h after infection we observed robust genome replication in all viruses, with all mutants lagging in genome replication behind *dl*309 expressing wt E1A. This trend continued at 72 h also and at this point all mutant viruses still lagged behind *dl*309 virus. Interestingly, *dl*311 was not the worst virus in terms of genomes replicated despite growing very poorly. Ultimately, the mutant viruses did not replicate their genomes as efficiently as wt E1A expressing virus, with some mutants lagging by as much as 100-fold in genomes per cell (such as *dl*1135).

### 3.5. Effects of E1A C-Terminus Mutations on S-Phase Induction

**Figure 7:**
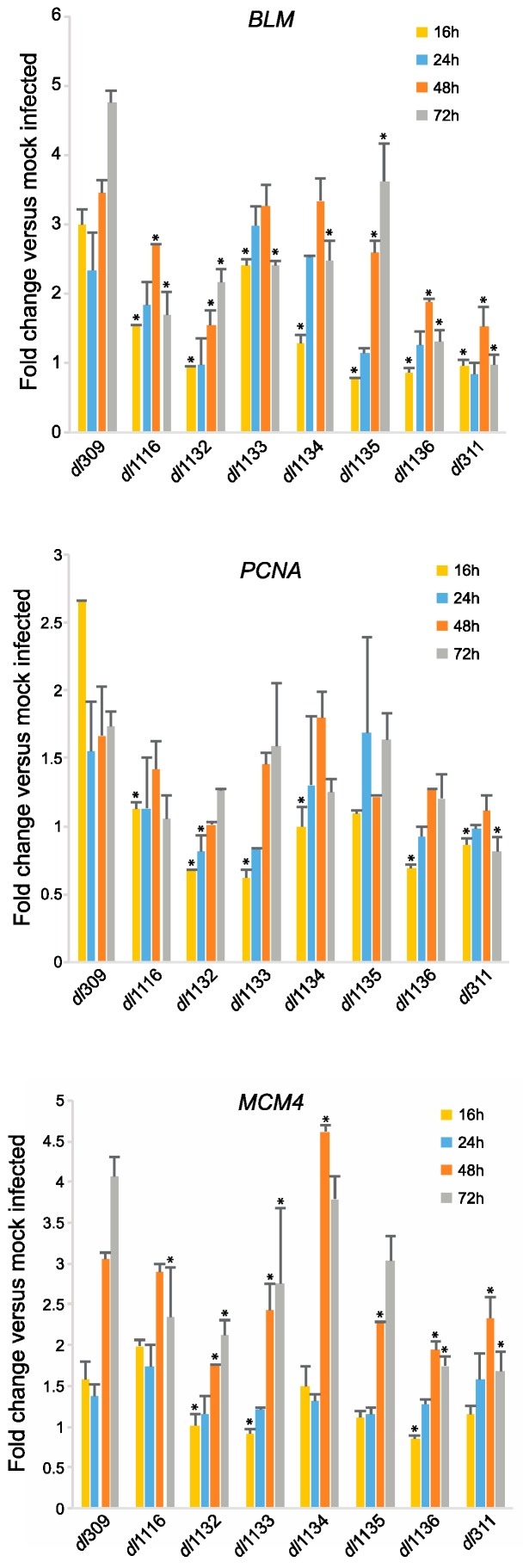
C-terminus deletions in E1A affect induction of cell cycle genes in arrested cells. Arrested WI-38 cells were infected with the indicated mutant viruses at a MOI of 100. At the indicated time points, total RNA was extracted using the TRIzol reagent and treated with DNase I. RNA was converted to cDNA using VILO Master Mix reverse transcriptase and relative levels of the indicated cellular mRNAs were quantified by qPCR using BioRad CFX96 with Applied Biosystems SYBR Master Mix for CFX. Expression levels were compared to those obtained from mock-infected cells using the Pfaffl method with GAPDH as reference. Statistically significant changes as compared to genes expressed in *dl*309-infected cells are indicated with an asterix (*), others are not significant. For statistically significant differences, the *p* values are as follows: *BLM*—*p* ≤ 0.0001; *PCNA*—*p* ≤ 0.0132; *MCM4*—*p* ≤ 0.0003. Error bars represent standard deviation of biological replicates, *n* = 3.

To determine how the different E1A mutants impact cellular reprogramming of arrested cells we examined induction of S-phase specific genes that were previously shown to be regulated by E1A [24]. We looked at expression of *Bloom* (*BLM*), *Proliferating Cell Nuclear Antigen* (*PCNA*) and *Mini-Chromosome Maintenance 4* (*MCM4*) at 16, 24, 48 and 72 h after infection as compared to mock infected cells (Figure 7). At 16 h after infection, compared to the mutants, levels of BLM mRNA were highest in cells infected with *dl*309, expressing wt E1A. *dl*1133 had the second highest BLM mRNA levels, while *dl*311, *dl*1135 and *dl*1136 had the lowest levels at 16 h after infection. This trend continued later into the infection particularly with *dl*311 and *dl*1136, which never induced *BLM* expression beyond 2-fold over mock infected cells. Interestingly, *dl*1135, which had very low levels of induction of *BLM* at 16 h, was able to recover later, particularly at 48 and 72 h after infection. *dl*1116 and *dl*1132 were also relatively poor at inducing *BLM* expression, whereas *dl*1133 and *dl*1134, although not as good in induction as wt virus, had relatively high levels of BLM mRNA. Similar trends were observed for expression of *PCNA*, although overall this gene was only induced by 2.5-fold over mock infected cells at 16 h. The mutant viruses either did not induce *PCNA* expression at all, such as *dl*311 or induced it poorly, such as *dl*1132 and *dl*1136. Lastly, induction of *MCM4* lagged behind that of *BLM* and *PCNA* and correlated closely with initiation of viral DNA replication, with levels of MCM4 mRNA increasing at 48 h after infection in these cells. Interestingly, *dl*1134 induced this gene to the highest levels at 48 h after infection, slightly higher than *dl*309, before dropping off slightly at 72 h. Overall, however, the trend for *MCM4* was similar to what was seen for the other genes.

**Figure 8:**
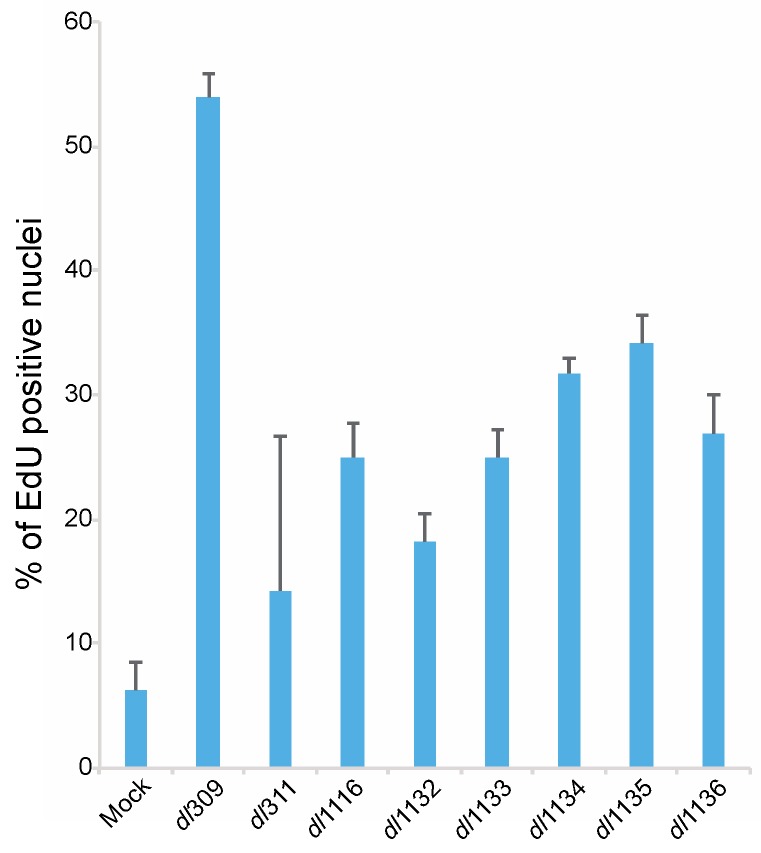
C-terminus of E1A is important for induction of DNA replication in arrested WI-38 cells. Arrested WI-38 cells were infected with the indicated viruses at a MOI of 100 for 23 h, pulsed with EdU for 1 h, fixed and stained for EdU using the Click-It EdU labeling kit. Cells were also labeled for E1A using the M58 antibody and anti-mouse Alexa594-conjugated secondary antibody. Cellular nuclei were also counterstained using DAPI. All mutants were significantly different from *dl*309 with a *p* ≤ 0.0341. Error bars represent standard deviation of biological replicates, *n* = 5.

We also determined how deletions within the C-terminus affect the ability of the virus to induce S-phase. To examine induction of DNA replication we assayed for EdU incorporation in arrested cells 24 h after infection (Figure 8). Mock infected cells showed a background level of DNA replication below 10% of total cells. HAdV *dl*309 virus expressing wt E1A induced S-phase in over 50% of cells, which is in line with previous reports [2,24], whereas all viruses expressing mutant E1A showed deficiencies in induction of S-phase. Of these mutants, *dl*311 was the poorest closely followed by *dl*1132. Induction of cellular DNA replication at 24 h hovered between 20% and 40% for all mutants and overall showed similar pattern to the induction of cellular S-phase specific genes (Figure 7).

## 4. Discussion

The present study examines the contribution of the C-terminus of E1A to adenovirus replication, growth, gene expression, protein expression, the modulation of cellular S-phase specific genes and induction of the cell cycle in arrested normal diploid lung fibroblasts. Viruses which had deletions in the C-terminus of E1A were found to be deficient for growth, protein and gene expression, genome replication and induction of S-phase. There was a large variability in phenotypes observed between the different E1A deletion mutants and predictably, the virus expressing E1A that was missing most of the region encoded by exon 2 (*dl*311) grew poorly and was severely deficient for induction of S-phase and S-phase specific genes.

The region of E1A encoded by the second exon of the gene has been implicated in a variety of cellular and viral processes via studies of mutants and binding partners [25]. Recently, we identified three novel C-terminus binding proteins that likely contribute to some of the observed phenotypes. RuvBL1, which binds in the region deleted in mutants *dl*1132 and *dl*1133, is involved in suppression of interferon stimulated gene activation via a direct interaction with E1A [11]. Mutants deleted for this region are unable to efficiently suppress interferon and part of the observed growth deficiency, particularly apparent at 96 h after infection, may be caused by loss of binding to RuvBL1. Similarly, interaction with DREF, which relies on residues missing in mutant *dl*1134, is important for innate antiviral response [9] and this may be compromised in this mutant as it also grows quite poorly. Lastly, Ku70, which E1A binds via residues 241–289, plays a role in suppression of the DNA damage response pathway [10] and deletions in this region affect many aspects of the viral life cycle. Furthermore, in addition to loss of binding to potential targets by E1A, some of the observed defects may be caused by alteration to protein conformation or spacing between regions of E1A.

Most of the functions of the C-terminus of E1A have been studied in the context of effects on cellular processes, with only a few studies looking at aspects of viral replication. For example, CtBP was identified as binding within CR4 of E1A and playing an important role in E1A-mediated transformation [5]. Further studies have shown that E1A can form a tri-partite complex with CtBP and ZNF217, enhancing ZNF217 binding to CtBP in order to alter the cellular transcriptional program [26]. FOXK1 was also identified as a C-terminus binding protein that is important in E1A mediated suppression of transformation [6]. Beyond the well-studied roles of E1A C-terminus in transformation [5,6,27,28,29,30], there is some evidence of this region playing a regulatory role in viral transcription [31]. Particularly, the region encoded by exon 2 of E1A was sufficient for induction of expression of DBP [31]. Our results show that most deletions within the C-terminus affect expression of DBP (Figure 3 and Figure 5) and this is particularly evident at 48 h after infection at the protein level. Levels of DBP mRNA were highly variable and we observed very low levels at 16 and 24 h after infection for all of the mutants, while *dl*309-infected cells showed robust mRNA and protein levels at 24 h. Interestingly, levels of DBP were quite robust in *dl*311-infected cells and overall DBP protein levels did not correlate well with virus growth. This suggests that the C-terminus of E1A may act as a transcriptional repressor for some viral genes, perhaps via an interaction and recruitment of a repressor to the viral promoters that E1A occupies [9,32]. However, this repression may be enhanced with certain mutants that may lose activator binding or lost with others that may lose repressor binding. Clearly, there is a hitherto unappreciated complexity in the role of E1A C-terminus in transcriptional regulation.

Initiation of viral DNA replication occurred between 24 and 48 h after infection for all mutants (Figure 6), this is similar to what has been previously reported in arrested IMR-90 cells [2] and arrested WI-38 cells [22] and significantly later than what was seen in transformed cell lines, which was reported to occur between 10 and 20 h after infection in HT1080 cells [9]. Thus, the delayed viral growth kinetics that we observe coincide with expectations based on existing literature for growth arrested cells. Our observations suggest that it is host factors, rather than levels of viral proteins, that regulate initiation of S-phase and DNA synthesis. As long as sufficient levels of viral proteins are made to initiate the cell cycle and facilitate viral DNA replication, duplication of genomes will commence. We observed a disparity in the total number of genomes produced at different time points after infection (Figure 6) and there was a correlation between the total number of genomes at the given time point and levels of DBP. This suggests that although host intrinsic factors regulate initiation of viral gene expression, it is viral factors, particularly those expressed from the E2 transcriptional region, that govern the efficiency and total accumulation of viral genomes. The cellular factors governing viral genome replication are likely the same ones that are induced during S-phase entry. Mutant viruses showed deficiencies in S-phase induction that paralleled levels of S-phase specific gene expression (Figure 7 and Figure 8). However, the overall accumulation of viral genomes was not related to S-phase induction, further strengthening the notion that total genome accumulation is more dependent on viral protein levels rather than cellular factors.

Interestingly, induction of cellular S-phase specific genes was highly variable amongst the mutants (Figure 7). It is generally thought that the deregulation of these genes occurs via disruption of E2F-pRb interactions by the CR1-CR2 regions of E1A [3,33] or direct activation via interaction of E1A with the E2F-DP complex at cellular promoters [24], so this result was unexpected. Although some mutants showed good induction of S-phase specific genes (such as *dl*1134), others induced these genes very poorly, such as *dl*1132 and *dl*1136. These results largely do not correlate with tumourigenicity of these mutations in the E1A243R protein [5] and rather may suggest that the C-terminus of E1A has unappreciated roles in cell cycle regulation via transcriptional control. This is of high interests and merits future investigation.

Our analysis of the mutant E1A sub-cellular localization showed that E1A is predominantly a nuclear protein even when its nuclear localization signal is compromised (Figure 4). For example, a complete deletion of the second exon from the *E1A* gene resulted in a protein that is predominantly nuclear. This may be due to E1A piggybacking on cellular factors that are nuclear; alternatively, the non-canonical E1A nuclear localization signals present within CR1 and CR3 of the protein may still drive its nuclear localization [12,34]. Of all the E1A mutants analyzed, *dl*1136 showed most cytoplasmic E1A, with *dl*311, *dl*1134 and *dl*1135 also showing some cytoplasmic E1A. These mutants delete or affect the C-terminus E1A NLS but retain other nuclear localization signals located within the region encoded by the first exon of the protein [34], suggesting that the C-terminus signal plays a major role in protein localization. Interestingly, we did not observe pronounced cytoplasmic localization of E1A as observed by others with green fluorescent protein (GFP)-fusion constructs [12]. This is likely due to the presence of the large GFP tag, which was absent in our virally-expressed mutants, combined with attenuation, via mutagenesis, of the strong E1A NLS at the C-terminus. Additionally, there is possible interference of the N-terminal GFP fusion with E1A binding partners at the N-terminus, many of which may carry E1A to the nucleus in the absence of a strong intrinsic NLS.

The poor growth of *dl*1136, next only to *dl*311, is difficult to explain using the metrics that we used to assess viral replication. This mutant expressed most viral proteins at levels comparable to other mutants that grew much better (Figure 3 and Figure 4) and it was capable of relatively efficient genome replication (Figure 6) but was poor at induction of CPE (Figure 2). It was also severely impaired for S-phase specific gene induction but not S-phase induction, suggesting that even minimal disruption of cell cycle genes by the virus is sufficient for S-phase entry, which may also be facilitated by expression of other viral genes, such as E4 products [35]. It is likely, that the disruption of nuclear localization of E1A *dl*1136 has a substantial effect on its nuclear functions, particularly those that affect host processes needed for efficient viral growth. This is evident from severe transformation deficiencies of a similar mutant (deleted only for the NLS but in E1A243R background) in rodent cells [19,36]. 

In conclusion, we have analyzed the influence of E1A C-terminus deletions on the fitness of the virus in arrested normal lung fibroblasts. Arrested cells represent the natural target of the virus and these studies provide important insight into how the different regions of the C-terminus of E1A contribute to viral growth. Importantly, we showed that deletions of the C-terminus affect viral gene expression, viral DNA replication and induction of S-phase. Interestingly, deletions outside of CR4 had significant effects on viral fitness, suggesting that yet unidentified proteins may be bound by E1A via those regions and contribute to viral replication. Lastly, our results suggest that E1A may play hitherto unknown roles in the late phase of the viral life cycle.
